# Advances in Diagnosis and Treatment of Acute and Chronic Heart Failure: A Comprehensive Review

**DOI:** 10.3390/jcm15020618

**Published:** 2026-01-12

**Authors:** Courtney R. Kenyon, Laura Van Wyk, Andrew Flom, Ramzi Ibrahim, Hoang Nhat Pham, Sofia Lakhdar, Momina Iftikhar, Mahmoud Abdelnabi

**Affiliations:** 1 Department of Cardiovascular Medicine, Mayo Clinic, Phoenix, AZ 85054, USA; 2 Department of Medicine, University of Arizona, Tucson, AZ 85719, USA

**Keywords:** heart failure, biomarkers, imaging modalities, guideline directed medical therapies, device therapies, artificial intelligence

## Abstract

Heart failure (HF) remains a major cause of morbidity and mortality worldwide, with its prevalence continuing to rise due to an aging population and the increasing burden of cardiometabolic diseases. Advances in understanding HF pathophysiology—neurohormonal, inflammatory, and metabolic mechanisms—have led to significant improvements in diagnosis and management, emphasizing earlier detection and patient-centered approaches. Novel biomarkers have the potential to enhance risk assessment beyond traditional natriuretic peptides. Imaging advances can enhance structural and functional assessment, enabling more accurate phenotyping, disease characterization, and risk stratification. Recent advances and real-world data have been used to implement and optimize guideline-directed medical therapy (GDMT) for HF to reduce HF hospitalizations and cardiovascular mortality across the spectrum of HF etiologies. Adjunctive therapies are reserved for select patients with persistent symptoms or high-risk features despite optimal GDMT. Device- and transcatheter-based interventions include established and emerging technologies that address persistent symptoms, structural abnormalities, and hemodynamic abnormalities despite optimal GDMT, thereby expanding treatment options for high-risk patients. Collectively, these advancements highlight a paradigm shift toward precise, personalized approaches to HF management, thereby improving long-term outcomes across the spectrum of HF etiologies.

## 1. Introduction

### 1.1. Epidemiology of Heart Failure

The incidence of heart failure (HF) in the United States has generally declined or stabilized in recent years, particularly among older adults, with Medicare data showing a decrease from 36 to 27 cases per 1000 beneficiaries between 2011 and 2014, remaining stable through 2016. However, the absolute number of HF cases and hospitalizations continues to rise, driven by population aging and improved survival from other cardiovascular diseases. Notably, the incidence of heart failure with reduced ejection fraction (HFrEF) is decreasing, while heart failure with preserved ejection fraction (HFpEF) is increasing, reflecting shifts in risk factor profiles and improved recognition of HFpEF [1,2]. Disparities persist, with higher incidence and mortality among non-Hispanic Black populations in the US, and a growing burden among younger adults, likely related to rising obesity and diabetes rates. The lifetime risk of developing HF after age 45 ranges from 20% to 46%, varying by sex and race. Overall, while incidence rates may be stabilizing or declining in some populations, the global burden of heart failure is increasing due to demographic shifts, improved survival, and rising prevalence of risk factors [1,2,3].

### 1.2. Pathophysiology of Heart Failure

HF is a complex, multifactorial clinical syndrome caused by structural or functional cardiac abnormalities that impair ventricular filling or ejection, resulting in inadequate tissue perfusion and/or elevated intracardiac pressures. Common etiologies include ischemic heart disease, hypertension, valvular disorders, and cardiomyopathies [1]. An initial cardiac insult—such as myocardial infarction or chronic pressure overload—reduces cardiac output and activates compensatory neurohormonal pathways, particularly the sympathetic nervous system (SNS) and renin–angiotensin–aldosterone system (RAAS) [4,5,6]. Although initially adaptive, chronic activation becomes maladaptive, driving fluid retention, vasoconstriction, myocardial hypertrophy, fibrosis, apoptosis, and progressive ventricular dysfunction. Mitochondrial dysfunction, oxidative stress, inflammation, and endothelial dysfunction further contribute to disease progression [5,7,8].

HF is categorized by left ventricular ejection fraction into heart failure with reduced ejection fraction (HFrEF, LVEF ≤ 40%), heart failure with mildly reduced ejection fraction (HFmrEF, LVEF 41–49%), heart failure with preserved ejection fraction (HFpEF, LVEF ≥ 50%), and heart failure with improved ejection fraction (HFimpEF, previous LVEF ≤ 40% with a subsequent measurement > 40%) [1]. HFrEF is characterized by impaired contractility, progressive ventricular dilatation, and adverse remodeling driven by neurohormonal dysregulation. HFmrEF is a dynamic intermediate phenotype that can improve or worsen. HFpEF is characterized by preserved systolic function with impaired diastolic relaxation, commonly in the setting of systemic comorbidities such as hypertension, obesity, and diabetes, which promote microvascular dysfunction, inflammation, and myocardial stiffening [1,9]. This pathophysiologic heterogeneity necessitates a multifaceted diagnostic and therapeutic approach. Recent advances in molecular biology, cardiovascular imaging, pharmacology, and digital health have substantially transformed HF management, enabling more precise phenotyping and individualized treatment strategies [10,11,12,13].

### 1.3. Emerging Biomarkers in Heart Failure

While natriuretic peptides such as B-type natriuretic peptide (BNP) and N-terminal pro-BNP (NT-proBNP) and troponins remain essential for the diagnosis and risk stratification of HF, recent studies have identified a range of novel biomarkers that have the potential to provide complementary information on myocardial stress, injury, inflammation, and remodeling [14]. These biomarkers have the potential to refine diagnostic precision, enhance prognostic assessment, and support individualized therapy [14,15]. Emerging biomarkers include soluble suppression of tumorigenicity-2 (sST2), growth differentiation factor-15 (GDF-15), galectin-3 (Gal-3), soluble urokinase-type plasminogen activator receptor (suPAR), mid-regional pro-adrenomedullin (MR-proADM), cystatin C (CysC), high-sensitivity cardiac troponins (hs-cTn), heart-type fatty acid–binding protein (H-FABP), and vascular cell adhesion molecule-1 (VCAM-1) [15,16,17,18,19,20,21,22,23]. Among these, sST2 and Gal-3 are strongly associated with myocardial fibrosis and systemic inflammation and provide incremental prognostic value beyond natriuretic peptides in both acute and chronic HF [1,15,18,20,22]. GDF-15 reflects oxidative stress and myocardial inflammation and demonstrates strong mortality prediction, in some settings exceeding natriuretic peptides in acute decompensated HF [15,17,20]. Neurohumoral markers such as MR-proADM and copeptin, as well as renal and inflammatory markers including CysC and suPAR, further improve risk stratification [19,20,23]. Inflammatory cytokines (IL-6, TNF-α) correlate with symptom burden, adverse remodeling, and poor outcomes [20,24,25]. Additionally, free light chain immunoglobulins (FLCs) have emerged as independent predictors of mortality and rehospitalization across HF etiologies, including ischemic, non-ischemic, and myocarditis-related HF [26,27,28,29]. Recent studies support the use of multimarker strategies and the integration of omics-based approaches to enhance risk prediction and personalize HF management (Table 1) [16,21,23]. However, despite their potential, their routine clinical use remains evolving, and natriuretic peptides and high-sensitivity troponins remain the most validated for diagnosis and prognosis [20,30].

### 1.4. Biomarkers in Chronic Kidney Disease and Heart Failure

In patients with chronic kidney disease (CKD) and HF, cardiac biomarkers are essential for risk stratification and management, although interpretation is complicated by reduced renal clearance. hsTnT and NT-proBNP remain the primary biomarkers, with elevated levels strongly associated with incident HF, major adverse cardiovascular events, and mortality in CKD, independent of traditional risk factors and renal biomarkers. Serial measurements and CKD-specific cutoffs improve their prognostic accuracy, as both markers rise with declining glomerular filtration rate (GFR) [31,32,33,34,35,36]. BNP and fibroblast growth factor 23 (FGF23) also provide incremental prognostic value, with BNP reflecting myocardial stretch and FGF23 being independently associated with incident HF in CKD. sST2 and GDF-15 are markers of fibrosis and inflammation; sST2 is less affected by renal function and, when combined with NT-proBNP, improves diagnostic accuracy for HF in CKD. Galectin-3 is associated with adverse outcomes and mortality, but its independent prognostic value in CKD is less robust than other markers [37,38,39]. The American Heart Association (AHA) recommends that natriuretic peptides (BNP, NT-proBNP) and troponins be used for diagnosis, prognosis, and risk stratification in HF. Still, higher baseline levels in CKD require adjusted interpretation [38]. Combining multiple biomarkers (e.g., NT-proBNP, hsTnT, FGF23, BNP) with clinical risk models enhances risk prediction and reclassification in CKD populations [37].

### 1.5. Emerging Imaging Modalities in Heart Failure

Recent advances in multimodality imaging for diagnosing and managing acute HF have significantly enhanced the ability to provide comprehensive structural, functional, and tissue characterization. Emerging modalities include (Summarized in Table 2):

## 2. Point-of-Care Ultrasound (POCUS)

Point-of-care ultrasound (POCUS) has emerged as an essential diagnostic tool in the acute evaluation and management of HF. The routine use of POCUS, including focused cardiac ultrasound (FoCUS) and lung ultrasound (LUS), is recommended in the evaluation and management of acute HF syndromes. FoCUS provides a rapid bedside assessment of ventricular function with a reported sensitivity and specificity of 80.6%, while LUS demonstrates superior accuracy for detecting pulmonary congestion compared with chest radiography and natriuretic peptides, with pooled sensitivities and specificities ranging from 82.5% to 88.6% and 83.2% to 92.7% across meta-analyses. The presence of B-lines is an independent predictor of acute HF and outperforms standard diagnostic strategies [40]. Randomized data show that adding LUS to clinical evaluation significantly improves diagnostic accuracy for acute decompensated HF (AUC 0.95 vs. 0.87 with conventional approaches) and reduces diagnostic errors [41]. Serial assessment of B-lines and inferior vena cava diameter correlates with clinical response to diuretic therapy and informs hospitalization decisions [42]. POCUS-guided volume assessment also outperforms clinical examination and natriuretic peptides for diuretic adjustment, even when performed by non-physician providers [43]. The European Society of Cardiology (ESC) consensus further endorses POCUS for early dyspnea evaluation and therapeutic monitoring in acute HF, highlighting its feasibility, rapid learning curve, and high interobserver reliability [44,45]. Collectively, these data establish POCUS as a frontline tool for rapid, accurate, and adaptive management of acute HF.

## 3. Echocardiogram

Echocardiography remains the first-line imaging modality in HF due to its accessibility and ability to assess ventricular function, hemodynamics, and valvular disease. Advanced techniques, including speckle-tracking strain echocardiography (STE) and three-dimensional echocardiography (3DE), now allow more precise quantification of myocardial deformation and chamber volumes, improving early detection of dysfunction, phenotypic classification, and longitudinal monitoring [46,47,48].

## 4. Speckle Tracking Strain Echocardiography

STE plays an increasingly important role in both the diagnosis and management of HF. Global longitudinal strain (GLS) is more sensitive than left ventricular ejection fraction (LVEF) for detecting early or subclinical systolic dysfunction, including in HFpEF. Impaired GLS predicts adverse outcomes, supports phenotyping, and refines risk stratification across the HF spectrum [49,50,51]. Current guidelines endorse echocardiography as the preferred initial imaging modality and recognize myocardial deformation indices as valuable for identifying subclinical dysfunction, predicting HF development, differentiating HF phenotypes, and guiding advanced therapies such as cardiac resynchronization [1,52,53,54]. Serial strain assessment further enables objective monitoring of therapeutic response and early detection of myocardial injury, including chemotherapy-related cardiotoxicity [51,52].

## 5. Three-Dimensional Echocardiography

3DE plays an increasingly important role in the diagnosis and management of HF by providing more accurate and reproducible quantification of cardiac chamber volumes, ejection fraction, and valvular anatomy compared with conventional two-dimensional echocardiography (2DE). Current guidelines recommend echocardiography as the preferred initial imaging modality for HF evaluation, with 3DE offering superior accuracy for left ventricular (LV) volumes and ejection fraction, especially in patients with complex geometry or significant remodeling [1,55,56]. 3DE enables comprehensive assessment of LV and right ventricular (RV) size and function, left atrial volumes, and valvular anatomy, which are essential for HF diagnosis, risk stratification, and therapeutic decision-making. Its improved spatial resolution and geometric accuracy reduce reliance on geometric assumptions inherent to 2DE and significantly decrease interobserver variability, facilitating consistent longitudinal follow-up in HF clinics and more reliable assessment of remodeling and response to therapy [1,51,57]. This is especially important when evaluating candidacy for advanced device therapies such as cardiac resynchronization, where precise quantification of chamber volumes and mechanical dyssynchrony directly informs patient selection and optimization [1,51,57]. Advanced 3DE techniques, including 3D-STE, provide a comprehensive evaluation of myocardial mechanics by simultaneously assessing longitudinal, circumferential, radial, and area strains. Compared with 2D strain, 3D-STE demonstrates stronger correlations with myocardial fibrosis and LV dysfunction and offers improved efficiency, reproducibility, and reduced analysis time. Clinically, 3D-STE enables earlier detection of subclinical myocardial dysfunction and more accurate prognostication, particularly in conditions such as dilated cardiomyopathy, ischemic heart disease, and chemotherapy-induced cardiotoxicity [58,59,60]. Collectively, these advances position 3DE as a cornerstone modality for precise phenotyping, risk stratification, and personalized management across the spectrum of HF [48,61].

## 6. Cardiac Computed Tomography (CT)

Recent advances in cardiac computed tomography (CT), particularly coronary CT angiography (CCTA) and dual-energy CT (DECT), have significantly expanded its role in HF evaluation by enabling comprehensive anatomical assessment, noninvasive coronary evaluation, analysis of ventricular function, and characterization of myocardial tissue [62,63]. Cardiac CT is recommended when echocardiography is inadequate or when additional structural data is required [1,64]. CCTA is recommended in patients with new-onset HF or unclear etiology as a non-invasive modality for coronary artery disease (CAD) evaluation [1]. CCTA is especially valuable in patients with low-to-intermediate pretest probability of CAD due to its high negative predictive value, reducing the need for invasive coronary angiography [11,53,62,65,66]. Recent studies demonstrate that CT-based strategies effectively exclude obstructive CAD and uncover occult disease in a substantial proportion of HF patients (≈32% in newly diagnosed HFrEF) [62,65,67]. DECT further enhances HF evaluation by enabling noninvasive assessment of myocardial fibrosis, scar, and extracellular volume (ECV), with diagnostic performance approaching that of late gadolinium enhancement (LGE) on cardiac magnetic resonance (CMR) [68,69,70]. DECT is particularly useful when CMR is contraindicated and provides quantitative ECV mapping that correlates with adverse outcomes in both HFrEF and HFpEF, including increased hospitalization and mortality risk [68,69,70,71,72]. Although DECT offers improved spatial resolution for myocardial viability assessment, its lower contrast-to-noise ratio compared with CMR remains a limitation, and emerging multi-energy CT and iodine-mapping techniques show promise for improvement. When combined, CCTA and DECT offer a comprehensive, safe, and effective initial diagnostic strategy for evaluating both ischemic and non-ischemic etiologies of HF [67].

## 7. Cardiac Magnetic Resonance Imaging

Recent advances in CMR have established it as a central modality for HF diagnosis and management. CMR is recommended for accurate and reproducible assessment of cardiac volumes, masses, and systolic function, especially when echocardiography is inconclusive or insufficient, with the added advantage of superior anatomic resolution and lack of radiation exposure [1]. CMR uniquely enables noninvasive myocardial tissue characterization using LGE and quantitative mapping techniques, allowing detection of myocardial injury and fibrosis, differentiation between ischemic and nonischemic etiologies, and identification of specific cardiomyopathies, including myocarditis, sarcoidosis, amyloidosis, and iron overload [1]. The presence, pattern, and extent of LGE provide powerful prognostic information and guide decisions regarding device therapy and immunosuppression [73,74]. Quantitative T1, T2, and T2* mapping now permit precise assessment of diffuse fibrosis, edema, and iron deposition, supporting early diagnosis, refined phenotyping, and risk stratification, particularly when biopsy is not feasible [73,74,75]. In HFpEF, CMR offers a comprehensive evaluation of diastolic function, ventricular and atrial remodeling, and myocardial–pericardial pathology, surpassing echocardiography in diagnostic accuracy for certain etiologies [76]. Recent technological innovations, including accelerated imaging protocols, four-dimensional flow imaging, and artificial intelligence–assisted postprocessing, have improved efficiency, image quality, and accessibility, even in patients with arrhythmias or implanted devices, enabling serial monitoring of disease progression and therapeutic response [53,77]. Collectively, these advances position CMR as a cornerstone tool for precision phenotyping and personalized management across the spectrum of HF [53].

## 8. Nuclear and Molecular Imaging

Recent advances in nuclear and molecular imaging have expanded its role in HF, focusing on improved phenotyping, risk stratification, and the development of personalized therapeutic strategies. Modern PET (Positron Emission Tomography) and SPECT (Single Photon Emission Computed Tomography) techniques enable evaluation of myocardial perfusion, metabolism, inflammation, innervation, and amyloid deposition. Established clinical applications include fluorodeoxyglucose (FDG-PET) for inflammatory cardiomyopathies (e.g., sarcoidosis), Tc-99m pyrophosphate (PYP) imaging for transthyretin amyloidosis, and Iodine-123 Metaiodobenzylguanidine (I-123 MIBG) SPECT for sympathetic innervation, all of which provide important prognostic and therapeutic guidance in selected HF phenotypes [78,79,80,81]. Quantitative PET assessment of myocardial blood flow and flow reserve further supports evaluation of microvascular dysfunction and myocardial viability, informing revascularization decisions in ischemic HF [78,79,81]. Emerging molecular tracers targeting myocardial fibrosis, metabolic remodeling, oxidative stress, and cellular senescence are in active clinical development and hold promise for earlier diagnosis, therapy monitoring, and prediction of adverse remodeling [80,81]. Integration of these techniques through hybrid PET/CT and PET/MR platforms enhances diagnostic precision by combining molecular, functional, and structural information. Collectively, these advances reflect a shift toward imaging-driven, mechanism-based phenotyping that supports precision cardiology in HF [78,79,80,81].

## 9. Vortex Dynamics Imaging

Vortex dynamics imaging is an emerging echocardiographic and MRI-based technique that quantifies intracardiac flow patterns, providing novel insight into LV mechanical efficiency and HF pathophysiology. In HF—particularly HFrEF—vortex formation becomes fragmented and reduced in size, with increased diastolic energy loss and impaired mechanical efficiency. These abnormalities correlate with worse clinical status and adverse outcomes, supporting the use of vortex parameters as sensitive markers of disease severity and prognosis [82,83,84,85]. Quantitative indices such as kinetic energy fluctuation (KEF) and vortex strength independently predict major adverse cardiac events and add incremental prognostic value beyond conventional echocardiographic parameters [83,84]. Advanced modalities, including vector flow mapping, contrast echocardiography, and 4D-flow MRI, enable reproducible assessment of these flow dynamics in both research and clinical settings [86,87,88,89]. Although not yet incorporated into routine guidelines, accumulating evidence supports the use of vortex dynamics imaging as a valuable noninvasive adjunct for refined risk stratification and comprehensive evaluation of LV function in HF [48,89].

**Table 2:** Summary of Emerging Imaging Modalities in Heart Failure.

Modality	Key Features	Clinical Utility	Supporting Evidence/Guidelines	Limitations
Point-of-Care Ultrasound (POCUS)	- Rapid bedside assessment (FoCUS, LUS) - B-lines for pulmonary edema - IVC assessment	- Acute HF diagnosis - Volume status - Diuretic guidance	- AC, ESC, HFA recommend POCUS- AUC up to 0.95 with LUS - RCTs/meta-analyses support superior accuracy to X-ray/NPs	- Operator dependent - Requires training to avoid misdiagnosis
Echocardiography	Speckle Tracking (GLS): detects subclinical dysfunction 3D TTE: accurate chamber/valve assessment	- HF phenotyping - Risk stratification - Therapy monitoring - CRT candidacy	- ACC/AHA/HFSA recommend echo with GLS for early detection- 3D echo offers superior volume/EF accuracy	- GLS not a substitute for standard parameters - Limited by image quality
Cardiac CT	- CCTA for CAD - DECT for fibrosis/ECV - High negative predictive value	- Rule out CAD - Myocardial tissue characterization - Alternative to CMR	- ACC/AHA/HFSA recommend when TTE is suboptimal - ACR supports CCTA in unknown HF etiology	- Radiation exposure - Limited tissue contrast compared to CMR
Cardiac MRI (CMR)	- LGE, T1/T2 mapping - High spatial resolution - No radiation	- Tissue characterization - HF phenotyping - Prognostication - Iron overload/fibrosis detection	- ACC/AHA/HFSA Class IIa for HF diagnosis - Quantitative mapping validated histologically	- Limited availability - Contraindications (e.g., devices, renal failure)
Nuclear and Molecular Imaging	- PET/SPECT with specific tracers (FDG, PYP, MIBG) - Molecular targets: fibrosis, inflammation, metabolism	- Phenotyping - Risk stratification - Therapy monitoring - Amyloid, sarcoid, ischemia evaluation	- Supported for select indications (e.g., ATTR, sarcoid) - Hybrid PET/CT/MR improves precision	- Limited tracer availability - Radiation exposure - Cost
Vortex Dynamics Imaging	- Quantifies LV flow patterns (vortices, KEF) - Assessed via echo or 4D-MRI	- LV function assessment - Prognostication - Risk stratification	- Correlates with HF severity and outcomes - Reproducible with modern imaging	- Not yet guideline-endorsed - Limited clinical adoption

ACC: American College of Cardiology, ACEP: American College of Emergency Physicians, AHA: American Heart Association, AUC: Area Under the Curve, CAD: Coronary Artery Disease, CMR: Cardiac Magnetic Resonance Imaging, CRT: Cardiac Resynchronization Therapy, CCTA: Coronary Computed Tomography Angiography, DECT: Dual-Energy Computed Tomography, ECV: Extracellular Volume, ESC: European Society of Cardiology, FoCUS: Focused Cardiac Ultrasound, GLS: Global Longitudinal Strain, HFA: Heart Failure Association, HF: Heart Failure, HFpEF: Heart Failure with Preserved Ejection Fraction, HFrEF: Heart Failure with Reduced Ejection Fraction, IVC: Inferior Vena Cava, KEF: Kinetic Energy Fluctuation, LGE: Late Gadolinium Enhancement, LUS: Lung Ultrasound, LV: Left Ventricle/Left Ventricular, LVEF: Left Ventricular Ejection Fraction, MRI: Magnetic Resonance Imaging, NPs: Natriuretic Peptides, PET: Positron Emission Tomography, PYP: Technetium-99m Pyrophosphate, POCUS: Point-of-Care Ultrasound, RV: Right Ventricle/Right Ventricular, SPECT: Single-Photon Emission Computed Tomography, STE: Speckle Tracking Echocardiography.

## 10. Emerging Therapies in Heart Failure

### Pharmacological Therapies

Emerging pharmacological therapies for HF are being adopted or studied for their disease-modifying potentials that extend beyond current guidelines’ recommended indications (Summarized in Table 3).

## 11. Angiotensin Receptor-Neprilysin Inhibitors (ARNIs)

Sacubitril/Valsartan is established as first-line therapy for patients with HFrEF (NYHA II–IV), with robust evidence demonstrating reductions in cardiovascular mortality and HF hospitalizations. Sacubitril/Valsartan is recommended as a Class I, Level of Evidence A over Angiotensin-Converting Enzyme inhibitors (ACEis) or Angiotensin Receptor Blockers (ARBs) based on evidence from PARADIGM-HF trial and subsequent studies showing significant reductions in sudden cardiac death (SCD), likely related to reverse remodeling and improved function [13,90]. Initiation is appropriate in both ambulatory and stabilized hospitalized patients, including those naïve to ACEis/ARBs, as supported by PIONEER-HF and TRANSITION [1,11,13,91]. In HFmrEF and HFpEF, the benefit of Sacubitril/Valsartan is more modest. Based on PARAGON-HF and pooled analyses, sacubitril/valsartan may reduce HF events and cardiovascular death, particularly in patients with LVEF below normal (≤60%), with greatest benefit in those with LVEF ≤ 57% and in women; however, recommendations remain weaker (Class IIb), and the absolute effect size is less profound than in HFrEF [10,11,92,93]. Beyond hemodynamic benefits, sacubitril/valsartan reduces ventricular arrhythmic burden, including sustained and nonsustained ventricular tachycardia, premature ventricular complexes, and appropriate implantable cardioverter-defibrillator (ICD) shocks, with the greatest effect observed in non-ischemic cardiomyopathy. These effects are attributed primarily to reverse remodeling and reduced myocardial fibrosis [94,95,96,97,98,99,100,101,102]. Improved LVEF may decrease primary-prevention ICD eligibility in some patients and increase biventricular pacing percentage in those receiving cardiac resynchronization therapy (CRT) [13,94,98]. Ongoing studies are evaluating optimal use in HFpEF, post-myocardial infarction HF, advanced HF phenotypes, and in combination with SGLT2 inhibitors [103,104,105]. Major safety considerations include hypotension and angioedema, with lower rates of hyperkalemia and renal dysfunction compared with ACEis/ARBs [10,11].

## 12. Sodium-Glucose Co-Transporter-2 Inhibitors (SGLT2i)

SGLT2is are now recommended for symptomatic HF across the entire ejection fraction spectrum (HFrEF, HFmrEF, HFpEF), regardless of diabetes status, to reduce HF hospitalizations and cardiovascular mortality. DAPA-HF, EMPEROR-Reduced, DELIVER, and EMPEROR-Preserved—demonstrate consistent benefit across phenotypes, with up to ~30% relative risk reduction in major composite outcomes and significant renoprotective effects [1,10]. Emerging evidence further supports early initiation in newly diagnosed HFrEF and during hospitalization, with clinical benefits observed within days to weeks. These agents are generally well tolerated, with minimal effects on blood pressure and glycemia, and exhibit pleiotropic benefits including improved myocardial energetics, reduced epicardial adiposity, modulation of inflammation, favorable remodeling, and improved diastolic function [106,107]. Beyond guideline-indicated populations, observational evidence suggests SGLT2i therapy is safe and potentially beneficial in advanced HF settings. In left ventricular assist device (LVAD) recipients, small studies and meta-analyses report improvements in ejection fraction, glycemic control, diuretic requirements, weight, and pulmonary pressures without significant adverse effects on renal function, hemodynamics, or device performance [108,109,110]. Similarly, limited observational data support SGLT2i use in cardiac transplant recipients with diabetes, demonstrating favorable metabolic effects and acceptable safety, though randomized data and long-term graft outcomes remain unavailable [111,112]. While current guidelines do not provide specific recommendations for hypertrophic, restrictive, or infiltrative cardiomyopathies, growing mechanistic and observational evidence suggests potential cardiovascular benefits beyond glycemic control, including improved diastolic function, reduced myocardial fibrosis, and favorable cardiac remodeling [113,114,115]. In hypertrophic cardiomyopathy (HCM) with diabetes mellitus, SGLT2i use has been associated with improved diastolic function, functional capacity, and reduced HF events and arrhythmic risk, supported by preclinical data demonstrating enhanced myocardial relaxation and calcium handling [116,117,118]. In transthyretin amyloid cardiomyopathy (ATTR-CM), multiple propensity-matched cohort studies and meta-analyses indicate reduced mortality, fewer hospitalizations, improved functional status, and lower arrhythmic burden, with consistent benefit across wild-type and hereditary disease and possible extension to amyloid light-chain (AL) amyloidosis [119,120,121]. Nonetheless, definitive randomized trials are needed before formal guideline recommendations can be extended to these specialized cardiomyopathy populations [1].

## 13. GLP-1 Receptor Agonists and Receptor Agonists (GLP-1/GLP-1RAs)

Recent trials and meta-analyses indicate that GLP-1RAs improve clinical status in HFpEF, particularly among patients with obesity or diabetes. Although they do not reduce mortality, GLP-1RAs significantly decrease HF hospitalizations and improve symptoms, functional capacity, and quality of life. These benefits appear to be mediated primarily through weight loss, improved endothelial function, reduced inflammation, favorable metabolic effects, and decreased epicardial adiposity, rather than through direct inotropic or myocardial remodeling effects [122,123,124]. In contrast, GLP-1RAs have not demonstrated improvement in left ventricular function or clinical outcomes in HFrEF and may increase heart rate [124,125]. In patients with HF and diabetes, GLP-1RAs remain appropriate for glycemic control, with additional cardiometabolic benefits, including weight reduction, blood pressure lowering, lipid improvement, and reduced atherosclerotic events. However, they should not be prescribed with the primary goal of improving HF outcomes [126]. Current evidence supports their use to improve symptoms and reduce HF events in HFpEF with obesity or diabetes, while ongoing trials will further clarify their role across broader HF populations [127,128].

## 14. Ivabradine

Ivabradine is recommended as adjunctive therapy for patients with stable, symptomatic HFrEF (NYHA II–III, LVEF ≤ 35%) in sinus rhythm with resting heart rate ≥ 70 bpm despite maximally tolerated beta-blocker therapy to reduce HF hospitalization, with only a modest impact on cardiovascular mortality, as demonstrated in the SHIFT trial and subsequent meta-analyses [1,13]. Ivabradine provides additional heart rate control in patients who remain tachycardic or cannot tolerate higher beta-blocker doses, reflecting the established prognostic importance of heart rate in HFrEF [13]. Beyond hospitalization reduction, ivabradine improves surrogate markers, including LV function, reverse remodeling, and exercise capacity, although effects on quality of life are inconsistent [129,130]. Emerging data suggest that once-daily sustained-release formulations may offer comparable efficacy with improved adherence [131]. Preclinical and translational studies indicate potential pleiotropic effects—anti-remodeling, anti-fibrotic, and anti-inflammatory—which may expand its therapeutic role beyond heart rate reduction; however, further validation is required [132,133]. Ongoing trials aim to refine patient selection, assess long-term outcomes, explore use in acute decompensated HF, and integrate ivabradine within evolving heart failure pharmacotherapy strategies [133,134].

## 15. Finerenone

Finerenone is a nonsteroidal mineralocorticoid receptor antagonist that has emerged as a promising therapy for patients with HFmrEF and HFpEF. In the FINEARTS-HF trial, finerenone added to standard therapy significantly reduced the composite of total worsening heart failure events, driven primarily by fewer hospitalizations and urgent HF visits, although no significant reduction in cardiovascular mortality was observed in patients with LVEF ≥ 40% [135]. Benefits were consistent across key subgroups, including age, baseline ejection fraction, BNP levels, body mass index, and baseline health status, and were accompanied by modest but significant improvements in patient-reported symptoms as measured by the Kansas City Cardiomyopathy Questionnaire [136,137,138]. Pooled analyses from FIDELIO-DKD, FIGARO-DKD, and FINEARTS-HF further support reductions in cardiovascular death or HF hospitalization in HFmrEF/HFpEF populations, with a generally favorable safety profile except for increased hyperkalemia risk [139]. Further evidence is required to clarify its role across the full ejection fraction spectrum, optimal integration with contemporary guideline-directed medical therapy, effects on hard endpoints including cardiovascular and all-cause mortality, and safety in patients with advanced chronic kidney disease or high hyperkalemia risk [139,140,141]. Ongoing trials—including REDEFINE-HF, CONFIRMATION-HF, and FINALITY-HF—are expected to clarify these issues, including their potential efficacy in HFrEF and in broader, non-diabetic populations [142].

## 16. Omecamtiv Mecarbil

Omecamtiv mecarbil is a selective cardiac myosin activator developed to enhance systolic function in HFrEF without increasing myocardial oxygen demand. In the GALACTIC-HF trial, omecamtiv mecarbil reduced the composite endpoint of first heart failure event or cardiovascular death by 8% compared with placebo, without increasing myocardial ischemia, ventricular arrhythmias, or cardiovascular mortality [143]. However, the METEORIC-HF trial showed no significant improvement in exercise capacity, peak VO_2_, daily activity, or quality of life [144]. Meta-analyses confirm modest clinical benefit, including reduced stroke risk, but no significant effect on all-cause mortality, HF hospitalizations, or patient-reported quality of life [145]. Post hoc analyses indicate greater benefit in patients with advanced disease—NYHA III–IV symptoms, LVEF ≤ 30%, and recent HF hospitalization—with preserved hemodynamic and renal safety, supporting potential use in patients intolerant to neurohormonal blockade [146]. Although promising for select high-risk patients, omecamtiv mecarbil remains investigational and is not currently guideline-endorsed. Ongoing studies are evaluating optimal patient selection, long-term safety, and combination strategies with contemporary HF therapies [147,148,149].

## 17. Vericiguat

Vericiguat is an oral soluble guanylate cyclase (sGC) stimulator that enhances the impaired nitric oxide (NO)–sGC–cGMP pathway in HFrEF, leading to improved endothelial function, reduced fibrosis, and vasodilation without increasing myocardial oxygen demand [150,151]. In the VICTORIA trial, vericiguat reduced the composite endpoint of cardiovascular death or first heart failure hospitalization by 10% in high-risk HFrEF patients with recent clinical worsening [152]. Accordingly, current guidelines recommend vericiguat for select high-risk patients with recent worsening HFrEF despite GDMT to reduce hospitalizations and cardiovascular death [13]. Ongoing trials are evaluating its role in broader HFrEF populations and in HFpEF; however, early data in HFpEF show no significant improvement in functional capacity or exercise tolerance [153,154]. Prior studies suggest consistent reduction in HF hospitalization but less definitive effects on mortality, particularly in patients with very high NT-proBNP levels [155,156]. Future research should focus on refining patient selection, expanding indications, and integrating within comprehensive HF management strategies.

**Table 3:** Summary of Emerging Pharmacological Therapies in Heart Failure.

Therapy	Mechanism/Target	Key Evidence and Trials	Clinical Benefits	Populations with Evidence	Safety Considerations	Future Directions
ARNI (Sacubitril/Valsartan)	Neprilysin inhibition + RAAS blockade	PARADIGM-HF, PIONEER-HF, TRANSITION, PARAGON-HF	↓ CV mortality, ↓ HF hospitalizations, reverse remodeling, ↓ arrhythmic burden, ↑ CRT pacing percentage	Strong in HFrEF (Class I, LOE A); modest in HFmrEF/HFpEF (Class IIb), women, LVEF ≤ 57%	Hypotension, angioedema (lower hyperkalemia/renal dysfunction vs. ACEi/ARB)	Optimal use in HFpEF, combo with SGLT2i, post-MI and advanced HF
SGLT2 Inhibitors	Glucose and sodium reabsorption inhibition in proximal tubule; pleiotropic cardiac effects	DAPA-HF, EMPEROR-Reduced, EMPEROR-Preserved, DELIVER	↓ CV death/HF hospitalization (~30%), improved renal outcomes, benefits across EF spectrum, rapid onset of effect	HFrEF, HFmrEF, HFpEF (regardless of diabetes); observational evidence in LVADs, transplant, HCM, ATTR-CM	Generally well tolerated; mild BP reduction; minimal glycemic effects; careful in transplant/LVAD	Role in infiltrative/restrictive cardiomyopathies, ATTR-CM, upfront initiation in acute HF
GLP-1 Receptor Agonists	Enhances incretin signaling, improves metabolic/inflammatory profile	STEP-HFpEF (semaglutide), other trials/meta-analyses	↓ HF hospitalizations in HFpEF with obesity/diabetes, improved symptoms, QoL, functional capacity; weight loss, BP and lipid benefits	HFpEF + obesity/diabetes; no benefit in HFrEF	↑ HR possible, no mortality benefit, not HF disease-modifying	Ongoing trials to clarify role beyond obesity/diabetes
Ivabradine	If-channel inhibitor → ↓ HR in sinus rhythm	SHIFT trial	↓ HF hospitalization, modest ↑ LVEF, reverse remodeling; HR control when β-blockers inadequate	HFrEF (LVEF ≤ 35%), NYHA II–III, sinus rhythm HR ≥ 70 bpm, on GDMT	Bradycardia, phosphenes; no significant mortality benefit	Sustained-release formulations, broader phenotypes, pleiotropic (anti-fibrotic/anti-inflammatory) effects
Finerenone	Nonsteroidal mineralocorticoid receptor antagonist (MRA)	FINEARTS-HF; pooled analyses from FIDELIO-DKD, FIGARO-DKD	↓ Total HF events (hospitalizations/urgent visits) in HFmrEF/HFpEF, modest KCCQ symptom score improvement, pooled analyses suggest ↓ CV death or HF hospitalization	HFmrEF/HFpEF, diabetic kidney disease	Hyperkalemia risk; limited data in advanced CKD (eGFR < 25 mL/min/1.73 m^2^) or high hyperkalemia risk	Clarify role across EF spectrum, optimize GDMT combinations, define effect on CV/all-cause mortality
Omecamtiv Mecarbil	Selective cardiac myosin activator (↑ systolic function without ↑ O_2_ demand)	GALACTIC-HF, METEORIC-HF	↓ HF events/CV death (GALACTIC-HF), safe in severe HFrEF; no improvement in exercise capacity/QoL	Severe HFrEF (NYHA III–IV, LVEF ≤ 30%, recent hospitalization)	Generally safe; no effect on BP/renal function	Role in severe HF not tolerating GDMT; combination strategies; long-term outcomes
Vericiguat	Soluble guanylate cyclase stimulator → enhances NO–sGC–cGMP signaling	VICTORIA trial	↓ CV death or HF hospitalization in high-risk HFrEF; especially recent worsening HF	High-risk HFrEF with recent decompensation; less robust in very high NT-proBNP	Hypotension, syncope, anemia	Ongoing trials in HFpEF (VALOR, others); refining patient selection; expanded indications

HF: Heart Failure; HFrEF: Heart Failure with Reduced Ejection Fraction; HFmrEF: Heart Failure with Mildly Reduced Ejection Fraction; HFpEF: Heart Failure with Preserved Ejection Fraction; NYHA: New York Heart Association; LOE: Level of Evidence; CV: Cardiovascular; RAAS: Renin–Angiotensin–Aldosterone System; ACEi: Angiotensin-Converting Enzyme Inhibitor; ARB: Angiotensin Receptor Blocker; CRT: Cardiac Resynchronization Therapy; ICD: Implantable Cardioverter-Defibrillator; LVEF: Left Ventricular Ejection Fraction; SGLT2i: Sodium–Glucose Co-Transporter-2 Inhibitor; GLP-1RA: Glucagon-Like Peptide-1 Receptor Agonist; QoL: Quality of Life; LVAD: Left Ventricular Assist Device; HCM: Hypertrophic Cardiomyopathy; ATTR-CM: Transthyretin Amyloid Cardiomyopathy; AL: Amyloid Light-chain; GDMT: Guideline-Directed Medical Therapy; O_2_: Oxygen; NO: Nitric Oxide; sGC: Soluble Guanylate Cyclase; cGMP: Cyclic Guanosine Monophosphate; BP: Blood Pressure; MI: Myocardial Infarction; NT-proBNP: N-terminal pro-B-type Natriuretic Peptide, KCCQ: Kansas City Cardiomyopathy Questionnaire.

## 18. Device Therapies in Heart Failure

Device-based therapies are central to the management of HF, especially in patients with HFrEF who remain symptomatic despite optimized GDMT. ACC and other societies recommend the following device-based interventions for HFrEF.

### 18.1. Implantable Cardioverter-Defibrillators (ICDs)

ICDs are indicated for primary prevention in patients with LVEF < 35% despite ≥3 months of optimized GDMT and for secondary prevention following ventricular fibrillation or sustained ventricular tachycardia. Landmark trials, including MADIT II and SCD-HeFT, demonstrated significant reductions in all-cause mortality, particularly in ischemic cardiomyopathy, with less consistent benefit in nonischemic etiologies [1,11]. Subcutaneous ICDs are preferred in patients with high infection risk, limited vascular access, prior device infections, or younger age, but are unsuitable for those requiring pacing, antitachycardia pacing (ATP), or CRT [157]. Despite proven benefits, important limitations persist. Device-related complications include infection, lead failure, inappropriate shocks, and device malfunction. Inappropriate shocks—occurring in 5–15% of patients—are associated with psychological distress, reduced quality of life, myocardial injury, worsening ventricular function, and increased risk of HF hospitalization and mortality [158,159,160,161]. The absolute risk of SCD in HFrEF has declined with optimized GDMT, diminishing the incremental benefit of ICDs, particularly in nonischemic disease [162,163]. Survival benefit is further attenuated in older patients, severe comorbidities (e.g., CKD), or advanced HF (NYHA IV not eligible for advanced therapies); therefore, careful risk-benefit assessment is recommended in these high-risk populations [159].

### 18.2. Cardiac Contractility Modulation (CCM)

CCM is a device-based therapy that enhances myocardial contractility by delivering high-voltage, biphasic electrical impulses to the right ventricular septum during the heart’s refractory period. These impulses improve calcium handling, thereby enhancing the strength of contraction and reversing maladaptive gene expression without increasing myocardial oxygen consumption [159]. ACC and other societies recognize CCM as FDA-approved for patients with HFrEF (LVEF 25–45%) who remain symptomatic despite optimized GDMT and are not candidates for CRT, particularly those with QRS duration < 130 ms [159,164]. Clinical studies and meta-analyses demonstrate a statistically significant improvement in exercise tolerance (e.g., 6 min walk test), quality of life (Minnesota Living With Heart Failure Questionnaire), and NYHA functional class with CCM [165,166]. While improvements in peak oxygen consumption (VO_2_) have been observed, meta-analytic data suggest these may not always reach statistical significance [166]. CCM also facilitates reverse remodeling, with increases in LVEF and reductions in LV volumes [166,167]. Registry data indicate a substantial reduction in HF hospitalizations over two years following CCM implantation, with rates decreasing from 0.74 to 0.25 events per patient-year (*p* < 0.0001) [167]. Although CCM is primarily studied in HFrEF, ongoing research is evaluating its role in broader HF populations such as patients with HFmrEF or HFpEF [168,169]. Additionally, long-term outcome data on mortality are still limited, and further randomized trials are needed to refine patient selection and lead positioning, and incremental benefits in addition to GDMT to improve overall survival [1,166,167].

### 18.3. Baroreflex Activation Therapy (BAT)

BAT is a device-based treatment that electrically stimulates carotid baroreceptors to reduce sympathetic activity and enhance parasympathetic tone, primarily indicated for resistant hypertension and has received FDA approval for select patients with HFrEF who are not candidates for CRT [1,170]. BAT involves an implantable pulse generator with leads placed near the carotid sinus. This stimulation lowers heart rate, reduces cardiac workload, promotes vasodilation, and improves renal perfusion, leading to lower blood pressure and potential HF symptom relief [1,170]. The degree of stimulation is titrated to individual hemodynamic response. Adverse events are generally mild and manageable with device parameter adjustments [171]. While early trials had mixed efficacy results, long-term data support sustained blood pressure reduction and a favorable safety profile [172]. In patients with HFrEF, current evidence suggests that BAT is associated with improved exercise capacity (6 min walk distance), quality of life, and NT-proBNP reductions with BAT, though no significant effect on mortality or hospitalization rates has been established [1,172,173]. Symptom and functional improvements are durable over several years, and the safety profile remains favorable [171,174]. Future research should focus on evaluating its role in broader HF populations. Long-term outcome data on hard clinical endpoints (e.g., mortality, major adverse cardiovascular events) are lacking. Further studies are needed to determine its impact on cardiac remodeling, head-to-head comparisons with other neuromodulation or device therapies with emphasis on mechanistic studies to clarify inter-individual variability in response, refine patient selection and study its incremental benefits in addition to other therapies to improve overall outcomes [174,175].

### 18.4. Wearable and Implantable Sensors in Heart Failure Management

Wearable and implantable sensors play an increasingly important role in HF management by enabling remote monitoring, early detection of clinical deterioration, and potentially reducing hospitalizations and improving outcomes in selected patient populations. These devices may facilitate early detection of HF exacerbations, support patient engagement, and potentially guide management decisions to reduce HF hospitalization; however, their impact on long-term clinical outcomes such as all-cause mortality is not yet established, and further prospective studies are needed [13,176]. (Summarized in Table 4).

### 18.5. Implantable Pulmonary Artery Pressure Sensors

Implantable devices, such as pulmonary artery (PA) pressure sensors (e.g., CardioMEMS), can provide direct hemodynamic data that can identify pre-symptomatic congestion and allow for timely therapeutic adjustments. ACC and other societies stated that PA pressure monitoring with implantable devices (such as CardioMEMS) may be considered in selected patients with NYHA class III HF and a recent hospitalization, but its overall value is uncertain [1,10,13]. This recommendation is based on mixed evidence: the CHAMPION trial showed a significant reduction in heart failure hospitalizations in this population, including those with both preserved and reduced ejection fraction, but methodological concerns exist due to its nonblinded design and differential contact with study personnel, which may have introduced bias. Subsequent studies, such as GUIDE-HF, did not confirm a significant benefit in broader populations, and the impact of the COVID-19 pandemic further complicated interpretation of results [177,178]. Additionally, meta-analyses confirmed a reduction in HF hospitalizations with no significant impact on all-cause mortality [179,180]. However, a recent patient-level meta-analysis suggests a possible mortality benefit in patients with HFrEF, but this finding requires further validation given the limitations in study design and follow-up duration [180,181]. While aggregate data supports the use of PA pressure monitoring in carefully selected patients with recurrent hospitalizations and persistent symptoms, highlighting a low rate of device related complications and sensor failure, further evidence is needed for individualized assessment and ongoing research to clarify its role in broader heart failure populations [182,183,184].

### 18.6. Cardiac Implantable Electronic Devices

Cardiac implantable electronic devices (CIEDs)—including implantable cardioverter-defibrillators, cardiac resynchronization therapy devices, and pacemakers—are increasingly utilized for both arrhythmia management and advanced HF monitoring. These devices can continuously record and transmit data on arrhythmias, heart rate, thoracic impedance (a surrogate for pulmonary congestion), patient activity, and respiratory rate. Multiparametric algorithms, such as those implemented in OptiVol (Medtronic) and HeartLogic (Boston Scientific), integrate these parameters to predict the risk of HF decompensation and provide actionable alerts to clinicians [176,183,185,186,187]. The clinical utility of these systems lies in their ability to detect subclinical changes that precede overt HF exacerbation, enabling earlier intervention and potentially reducing HF hospitalizations [176,185,187]. However, while multiparametric monitoring improves sensitivity for detecting impending decompensation, specificity remains moderate, and optimal clinical pathways following device alerts are still under investigation [176,185]. The AHA and other societies have endorsed remote monitoring of CIEDs as a means to improve outcomes and reduce unnecessary clinic visits, emphasizing its role in timely recognition of arrhythmias and HF worsening [188].

### 18.7. Bioimpedance-Based Wearables

Bioimpedance-based wearables are noninvasive devices designed to measure cardiac output and stroke volume continuously. Studies have demonstrated that these devices can achieve clinical equivalence to Doppler echocardiography in monitoring cardiac activity with comparable accuracy to Doppler echocardiography for cardiac output and stroke volume measurements, supporting their potential utility in congestive HF management and remote hemodynamic monitoring [189,190,191]. However, accuracy may vary depending on patient population, device configuration, and clinical context. Some studies note that while bioimpedance methods can provide useful trending information, absolute values may be over- or underestimated compared to echocardiography, and performance may deteriorate under certain physiological conditions (e.g., postural changes, exercise) [192,193,194]. However, device-specific limitations and patient factors must be considered when interpreting results [189,190,191].

### 18.8. Chest Patches and Multisensor Wearables

These devices can measure heart rate, respiratory rate, activity, and thoracic impedance or lung fluid volume. Advanced devices integrate multiple sensors and utilize artificial intelligence to predict HF exacerbations, as demonstrated in the LINK-HF study, which showed sensitivity up to 88% and specificity up to 85% for detecting impending HF rehospitalization by a median of 6.5 to 8.5 days [176,183,195,196].

### 18.9. Photoplethysmography (PPG) and Electrocardiographic (ECG) Sensors

Used in wrist-worn or garment-based devices to monitor heart rate, rhythm, and pulse arrival time, which can be relevant for arrhythmia detection and volume status assessment [176,195]. Large-scale studies (e.g., Apple Heart Study, Fitbit Study) have validated the use of these devices for atrial fibrillation screening, with positive predictive values ranging from 84% to 99% [176,195,197].

### 18.10. Acoustic Sensors and Skin-Impedance Technologies

Acoustic sensors provide phonocardiograms for heart sound analysis, while skin-impedance sensors can assess fluid status, both of which are being integrated into wearable garments and patches [176,195,198,199].

**Table 4:** Summary of Wearable and Implantable Sensors in Heart Failure Management.

Device/Technology	Mechanism and Features	Key Evidence and Findings	Limitations/Evidence Gaps
Implantable Pulmonary Artery Pressure Sensors (e.g., CardioMEMS)	Direct hemodynamic monitoring of PA pressures to detect pre-symptomatic congestion and guide therapy	- CHAMPION trial: ↓ HF hospitalizations in NYHA III patients, including HFrEF and HFpEF. - GUIDE-HF trial: No significant benefit in broader populations; COVID-19 impacted results. - Meta-analyses: ↓ HF hospitalizations, no mortality benefit. - Patient-level meta-analysis: possible mortality benefit in HFrEF (needs validation).	- Benefit mainly in selected high-risk NYHA III patients. - Methodological concerns (nonblinded, differential contact). - Unclear role in broader HF populations. - Long-term mortality impact uncertain.
Cardiac Implantable Electronic Devices (CIEDs) (ICDs, CRT, pacemakers with monitoring functions)	Continuous monitoring: arrhythmias, HR, thoracic impedance (pulmonary congestion), activity, respiratory rate. Multiparametric algorithms (e.g., OptiVol, HeartLogic) integrate signals to predict decompensation.	- Improves early detection of subclinical deterioration. - Multiparametric algorithms increase sensitivity. - AHA and societies endorse remote monitoring for arrhythmias and HF recognition.	- Specificity remains moderate. - Optimal management pathways after device alerts not standardized.
Bioimpedance-Based Wearables	Noninvasive continuous monitoring of cardiac output and stroke volume. Comparable to Doppler echocardiography in some studies.	- Studies show clinical equivalence to Doppler echocardiography. - Useful for trending hemodynamic changes.	- Absolute accuracy variable; may under- or overestimate values. - Performance affected by postural changes, exercise, and patient/device factors.
Chest Patches and Multisensor Wearables	Sensors for HR, RR, activity, thoracic impedance, lung fluid volume. Some use AI to integrate signals.	- LINK-HF study: 88% sensitivity and 85% specificity for predicting HF rehospitalization 6.5–8.5 days in advance.	- Performance varies by device. - Implementation pathways in clinical care still being established.
Photoplethysmography (PPG) and Electrocardiographic (ECG) Sensors	Wrist/garment-based monitoring of HR, rhythm, pulse arrival time for arrhythmia detection and volume status.	- Apple Heart Study, Fitbit Study: validated for AF screening (PPV 84–99%).	- Mainly validated for AF; HF-specific role needs further study.
Acoustic Sensors and Skin-Impedance Technologies	Acoustic sensors: phonocardiograms (heart sounds). Skin impedance: fluid status assessment. Often integrated into patches/garments.	- Emerging evidence suggests a potential role in congestion monitoring.	- Limited validation in HF. - Need larger prospective studies for clinical outcomes.

HF: heart failure; HFrEF: heart failure with reduced ejection fraction; HFpEF: heart failure with preserved ejection fraction; NYHA: New York Heart Association functional class; PA; pulmonary artery; ICD: implantable cardioverter-defibrillator; CRT: cardiac resynchronization therapy; HR: heart rate; RR: respiratory rate; AF: atrial fibrillation; PPG: photoplethysmography; ECG: electrocardiogram.

## 19. Transcatheter Valvular Interventions in Heart Failure

Recent advances in transcatheter valvular intervention for HF include the widespread adoption of transcatheter edge-to-edge repair for functional mitral regurgitation (FMR), transcatheter aortic valve replacement (TAVR) for aortic stenosis (AS), and emerging transcatheter therapies for tricuspid regurgitation (TR) in high-risk surgical candidates or remain symptomatic despite optimal GDMT. Current guidelines recommend TEER for patients with symptomatic HF and severe secondary MR despite optimized GDMT, with suitable anatomy, LVEF 20–50%, and LV end-systolic diameter ≤ 70 mm [1,200]. This recommendation is based primarily on the COAPT trial, which demonstrated significant reductions in HF hospitalizations and all-cause mortality with TEER plus GDMT compared with GDMT alone, whereas MITRA-FR showed no benefit—highlighting the critical importance of patient selection and GDMT optimization [1,13]. The MATTERHORN trial further demonstrated noninferiority of TEER compared with surgery in high-risk patients with superior procedural safety and lower periprocedural risk [201,202]. Key evidence gaps remain regarding optimal patient selection, the role of TEER in moderate MR and HFpEF populations, long-term effects on reverse remodeling and QoL, post-procedural GDMT strategies, and outcomes in patients with advanced comorbidities [13,202].

TAVR is first-line therapy for severe symptomatic AS in HF patients regardless of surgical risk and is indicated for asymptomatic severe AS with LVEF < 50% [203,204]. Recent trials support expanding TAVR to lower-risk patients, earlier intervention, and potentially moderate AS with HF. TAVR produces rapid afterload reduction, reverse remodeling, improvement in LVEF and NYHA class, reduced BNP levels, and improved survival with low procedural risk even in advanced HF [205,206,207]. However, patients with acute HF prior to TAVR remain at high risk for adverse outcomes, underscoring the importance of timely intervention [208]. Remaining challenges include uncertainty regarding long-term valve durability (>5–6 years), expanding indications in bicuspid valves and moderate AS, lifetime management strategies including redo-TAVR and coronary re-access, and persistent disparities in real-world outcomes [209,210,211,212,213].

TR is highly prevalent in HF and strongly associated with morbidity, recurrent HF hospitalizations, and reduced QoL. Medical therapy, mainly diuretics, provides only symptomatic relief and does not address the underlying valve pathology. Surgical intervention for isolated TR carries high perioperative risk and is rarely performed, leaving many patients undertreated [214,215]. Transcatheter TV procedures—including TEER, annuloplasty, and valve replacement—have demonstrated significant improvements in NYHA class, QoL, and HF hospitalizations, with emerging data suggesting potential mortality benefit compared to medical therapy alone [214,216,217]. Device-specific therapies (TriClip, PASCAL) show high procedural success and low complication rates [203,215]. Optimal outcomes depend on careful patient selection, including anatomical suitability, right ventricular function, and end-organ status, with pre-procedural volume optimization essential [215,218]. Current evidence gaps include optimal timing of intervention, management of moderate TR, long-term durability, integration with GDMT, and broader applicability across diverse HF phenotypes [219,220].

## 20. Left Ventricular Assist Devices (LVADs)

Current guidelines on LVAD therapies emphasize careful patient selection, indications, appropriate timing, and meticulous perioperative management for LVAD therapy in advanced HF. Durable LVAD implantation is indicated as bridge to transplantation (BTT), destination therapy (DT) in non-transplant candidates, or less commonly as bridge to myocardial recovery in patients with potentially reversible cardiomyopathy [221,222]. Candidates typically have NYHA class IIIB/IV symptoms refractory to GDMT, often requiring inotropes or temporary mechanical circulatory support. INTERMACS profiles (1–3) guide urgency and risk stratification, while preoperative assessment of right ventricular function, renal and hepatic reserve, and frailty is essential [221,223,224,225]. LVAD therapy significantly improves survival, functional capacity, and QoL compared with medical therapy alone, though bleeding, infection, stroke, and RV failure remain major risks [226,227]. Third-generation centrifugal-flow devices (e.g., HeartMate 3, HeartWare HVAD) use magnetic levitation technology, reducing pump thrombosis and stroke while improving long-term outcomes, with reported 5-year survival of approximately 60–81%. However, major complications remain common, including bleeding, infection, stroke, and RV failure. Despite advances, driveline infections continue to affect ~20% of patients within the first year, though novel strategies such as cold atmospheric pressure argon plasma therapy show early promise in reducing infection rates [228,229]. Current evidence gaps include persistent complication burden, lack of universally accepted risk prediction models, and uncertainty regarding optimal timing of referral. Emerging innovations—including fully implantable LVAD systems (LionHeart 2000, Leviticus Cardio), transcutaneous energy transfer technologies, AI-driven pump control, and advanced biomaterials—aim to improve durability, mobility, and long-term outcomes, but require validation in large, multicenter trials [1,226,228,230,231].

## 21. Heart Transplantation

Heart transplantation is recommended for selected patients with advanced (stage D) HF who remain symptomatic despite optimized GDMT, device therapy, and surgical interventions, to improve overall survival and QoL after exclusion of contraindications and completion of comprehensive multidisciplinary evaluation [1,232]. Recent advances include donor pool expansion to include the use of hepatitis C virus (HCV)–positive donors facilitated by direct-acting antiviral therapy and donation after circulatory death (DCD), which increases organ availability without compromising short-term outcomes [233,234]. The use of older and higher-risk donors, as well as improved donor–recipient matching through predicted heart mass and advanced antibody detection, further broadens recipient eligibility [235,236]. Innovations in organ preservation and transport have advanced with ex vivo perfusion and normothermic techniques, allowing longer ischemic times and greater geographic reach, thus reducing waitlist mortality and improving graft function [234,235,236]. Immunosuppression is increasingly individualized, with novel agents (e.g., belatacept, daratumumab, IL-6–directed therapies, IgG endopeptidase) and non-invasive biomarker surveillance enabling earlier detection and management of rejection, while minimizing infection and late complications [234,237]. Allocation policy changes, including the 2018 United Network for Organ Sharing (UNOS) revision, prioritize sicker patients and have reduced waitlist mortality [1,237]. Mechanical circulatory support (MCS) plays an expanding role, with temporary devices used for acute stabilization and as bridge-to-decision or bridge-to-recovery strategies [238], while durable LVAD implantation improves survival, functional capacity, and QoL in selected NYHA class IV patients, achieving median survival beyond five years and enabling transplantation in nearly half of BT recipients [238,239]. Emerging technologies addressing organ shortages include xenotransplantation and tissue engineering—including genetically modified porcine hearts, stem cell–derived organoids, and bioprinting—hold promise for addressing persistent organ shortages and transforming the future of advanced HF therapies [234]. Current evidence gaps include that most survival and QoL data are derived from observational cohorts rather than randomized trials, limiting comparisons with durable LVAD therapy, particularly in older or comorbid populations [1,240,241]. The long-term effects of recent donor allocation policy changes on waitlist and post-transplant outcomes remain incompletely defined [1,237,241]. Prospective data are limited to guide optimal timing of listing and integration of frailty, comorbidities, psychosocial factors, and patient preferences into candidate selection [1,242]. Long-term complications—including cardiac allograft vasculopathy, malignancy, renal dysfunction, and chronic rejection—remain under-studied in increasingly older and more complex recipients [233,237,241]. Furthermore, greater representation of diverse sociodemographic groups and patients with systemic diseases is needed to improve generalizability of transplant outcomes [1].

## 22. Heart Failure Management in Special Populations

### Elderly Population

Comorbidities, polypharmacy, and frailty are highly prevalent in older patients with HF, particularly those ≥75 years, with frailty affecting up to 60% of this population [13,243,244]. These patients (≥75 years) are frequently excluded from major HF clinical trials, limiting evidence for optimal management. The presence of multiple comorbidities (e.g., chronic kidney disease, diabetes, cognitive impairment), polypharmacy, and frailty complicates pharmacokinetics and pharmacodynamics, increasing susceptibility to adverse drug events, drug–drug interactions, and functional decline [13,244,245]. Accordingly, guidelines recommend individualized treatment in older adults, with lower starting doses of GDMT, slow titration, and close monitoring, incorporating patient goals, functional status, and social factors [1,10,11,13,245,246,247]. SGLT2is and ARNIs demonstrate cardiovascular and renal benefits in elderly and frail HF patients, including improved LVEF, lower NT-proBNP, and reduced hospitalizations and mortality, although risks such as hypotension, renal dysfunction, and volume depletion are more pronounced [243,248,249]. Polypharmacy increases the risk of electrolyte disturbances, falls, and hypotension, making dose adjustment, deprescribing, and patient–caregiver education essential. Frailty strongly influences medication tolerance and outcomes; multidomain physical and cardiac rehabilitation significantly improve functional capacity, QoL, and rehospitalization rates in frail older HF patients [13,243,248,250,251,252].

## 23. Pregnancy and Peripartum Cardiomyopathy

Recent guidelines and registry data emphasize the importance of early recognition and individualized management of PPCM, given symptom overlap with normal pregnancy and other causes of acute HF. Current recommendations prioritize prompt diagnosis using echocardiography and natriuretic peptides to distinguish PPCM from preexisting cardiomyopathy, preeclampsia-related pulmonary edema, and pulmonary embolism [253]. Multidisciplinary care involving cardiology, maternal–fetal medicine, genetics, and neonatology is essential, particularly in severe presentations and in women considering future pregnancies [1,254,255]. Genetic counseling and testing are advised at diagnosis or preconception, especially in families with cardiomyopathy or SCD, as titin truncating variants account for up to 20% of PPCM cases [253,255]. Pharmacologic therapy during pregnancy must balance maternal benefit with fetal safety. ACEis, ARBs, ARNIs, MRAs, and SGLT2is are contraindicated, while hydralazine–nitrate combinations and metoprolol are preferred, with cautious diuretic use to avoid placental hypoperfusion [1,254]. In patients with LVEF < 30%, short-term anticoagulation is often considered [1]. Bromocriptine has demonstrated improved maternal outcomes and is endorsed by the ESC as part of the “BOARD” regimen for acute PPCM, particularly in patients with LVEF < 25% or cardiogenic shock, with mandatory prophylactic anticoagulation [1,256,257]. MCS is reserved for refractory cases and may serve as a bridge to recovery or transplantation, though transplant recipients have poorer prognosis than other cardiomyopathy etiologies [253,257]. Long-term management includes continuation of HF therapy, serial echocardiography, counseling regarding risks of subsequent pregnancy—especially with persistent LV dysfunction—and shared decision-making with ongoing contraceptive planning [253,255].

## 24. Heart Failure and Comorbidities

For patients with HF and multimorbidity, ACC and other societies recommend a multidisciplinary care and tailored approach to address overlapping pathophysiologies [10,13].

## 25. Diabetes Mellitus

SGLT2is are recommended as a first-line treatment for hyperglycemia in patients with diabetes mellitus (DM) who have HF or are at high risk of HF. SGLT2is have been shown to reduce major adverse cardiovascular events, including HF hospitalization and cardiovascular death, in patients with type 2 DM with SGLT2is’ benefits are consistent across various patient demographics, across HF subtypes, and are not solely related to their glucose-lowering effects with the absolute benefit is greatest in the first year of therapy and in those with higher baseline risk [13]. SGLT2is should be considered regardless of baseline glycemic control, with monitoring for genitourinary infections and rare diabetic ketoacidosis [135,258,259].

## 26. Chronic Kidney Disease (CKD)

HF and CKD are bidirectionally linked, complicating management. Current evidence supports the use of SGLT2is, ACEis/ARBs, and ARNIs in those with mild-to-moderate CKD (eGFR ≥ 20 mL/min/1.73 m^2^), with demonstrated reductions in HF hospitalizations, cardiovascular death, and kidney disease progression. However, most trials excluded patients with advanced CKD (stages 4–5), so data in this group are limited. Initiation of these agents may cause a modest, expected decline in eGFR, and subsequent discontinuation should be avoided unless there is progressive or severe renal dysfunction. Multidisciplinary care, including combined cardiology-nephrology clinics, has been associated with improved outcomes and patient compliance in this high-risk population [260,261].

## 27. Autoimmune Diseases

In autoimmune diseases such as systemic sclerosis, primary cardiac involvement is common and associated with poor prognosis; however, management of these patients is not well-defined due to limited trial data. While no disease-specific HF therapies exist, recent observational data suggest that immunosuppressive therapies may reduce myocardial inflammation and improve cardiac biomarkers in patients with newly diagnosed scleroderma-associated cardiac involvement, supporting its possible role for immunosuppression in selected cases, but larger prospective studies are needed [13]. These patients require a multidisciplinary approach, with close coordination between cardiology and rheumatology to address cardiovascular side effects of immunosuppressive therapies and overlapping pathophysiology to optimize outcomes in these high-risk HF patients [262,263]. HF management in special populations was summarized in Table 5.

## 28. Telehealth in Heart Failure

Telehealth is playing an expanding role in HF management through remote monitoring, virtual visits, and digital health interventions. Structured telephone, video visits, and remote monitoring of physiologic parameters are recommended to facilitate early detection of decompensation, improve medication adherence, and enable timely therapeutic adjustments [188,264,265]. Meta-analyses and randomized trials demonstrate reductions in mortality and heart failure hospitalizations with telemonitoring, although outcomes vary by strategy, with the greatest benefit observed when multi-parameter monitoring is combined with patient education and medication management [266,267,268]. The TIM-HF2 trial demonstrated this approach, showing improved outcomes with comprehensive telehealth integration, including symptom tracking and actionable feedback [265,269]. Guidelines provide a moderate recommendation (Class IIb, Level B-R) for telehealth in HF, reflecting heterogeneity in trial results and ongoing implementation challenges [2]. While invasive monitoring (e.g., pulmonary artery pressure sensors) may benefit selected patients, noninvasive telehealth remains the foundation. Overall, telehealth improves access, supports self-management, and may reduce adverse outcomes when appropriately integrated with guideline-directed medical therapy and tailored to patient needs.

### 28.1. Artificial Intelligence and Machine Learning in Heart Failure 

Artificial intelligence (AI) and machine learning (ML) have an increasingly important role in the diagnosis and management of HF, with AI/ML algorithms transforming the interpretation of clinical, imaging, laboratory, and wearable device data to improve diagnostic accuracy, risk stratification, and personalized management of HF patients [270].

### 28.2. Classification and Diagnosis

AI-enhanced electrocardiogram (AI-ECG) models outperform traditional risk scores in early HF detection, serving as digital biomarkers for subclinical disease. Deep learning (DL) combining ECG and novel biosignals improves diagnostic accuracy, especially in primary care. Machine learning (ML) has been used to identify HF patients from electronic records, classify HF phenotypes like HFpEF, and distinguish HF from other dyspnea causes. AI decision support now matches specialist-level accuracy, benefiting resource-limited settings. Non-invasive continuous monitoring using ML with photoplethysmography and ECG signals also shows strong diagnostic performance [271,272]. Guidelines highlight that AI/ML applied to ECGs can detect occult structural heart disease earlier than traditional testing and can be integrated into clinical workflows to scale expert-level interpretation which can be beneficial in resource-limited settings [195].

### 28.3. Management and Follow-Up

Current evidence demonstrates that AI/ML algorithms are increasingly utilized to guide the management of HF patients, predict adverse outcomes, guide therapy selection, and monitor disease progression. AI models incorporating myocardial perfusion imaging have demonstrated improved accuracy in predicting hospitalization for acute HF exacerbation compared to conventional clinical parameters. Additionally, integrating imaging, clinical, and stress test data with DL-generated calcium scores yields superior accuracy in detecting patients at risk for HF hospitalization, potentially enabling earlier intervention and improved outcomes [195,273]. AI-based pharmacotherapy applications are being developed to optimize drug selection, predict therapeutic efficacy, and identify adverse drug reactions and interactions. AI algorithms can facilitate detection of drug–drug interactions and support personalized medication titration, especially in the context of complex GDMT regimens [183,274]. Digital therapeutics—software-based interventions for medication management—are emerging as adjuncts to streamline titration and monitoring but require rigorous validation and oversight [183,274].

### 28.4. Prognosis

AI and ML have shown promise in predicting mortality and hospital readmission in HF patients. AI and ML models play a central role in predicting prognosis, including mortality and hospital readmission rates, for patients with HF and acute HF. Deep-learning (DL)-based algorithms such as the DAHF model demonstrated superior predictive accuracy for in-hospital and long-term mortality in AHF patients compared to traditional risk scores. Specifically, DAHF model outperforms the Get With The Guidelines-Heart Failure (GWTG-HF) score and the Meta-Analysis Global Group in Chronic Heart Failure (MAGGIC) score for 12- and 36-month mortality endpoints [275]. AI-based electrocardiographic biomarkers provided independent and robust prediction of outcomes in AHF. The QCG-Critical score derived from deep learning analysis of baseline ECGs was an independent predictor of in-hospital cardiac death and long-term mortality, even after adjustment for conventional risk factors, echocardiographic LVEF, and NT-proBNP levels, supporting the utility of AI-ECG as a novel, accessible digital biomarker for risk stratification [276]. Additionally, Neural networks and support vector machines (SVMs) contributed to risk stratification and individualized care by using high-dimensional clinical data to improve prediction of mortality and readmission [277,278].

While AI-guided management strategies in HF offer potential for improved risk prediction, early intervention, and personalized pharmacotherapy, safety concerns—including drug interactions, model reliability, and regulatory compliance—must be actively addressed through rigorous validation and standardized oversight [195,274].

## 29. Precision Medicine in Heart Failure

Precision medicine in HF management aims to tailor diagnostic, prognostic, and therapeutic strategies to the individual patient by integrating clinical, genetic, biomarker, and multi-omics data to address the marked heterogeneity in HF phenotypes and treatment responses, moving beyond the traditional “one-size-fits-all” paradigm [279,280,281]. Contemporary approaches leverage genomics, transcriptomics, proteomics, metabolomics, microbiomics, and emerging biomarkers to enable deep phenotyping, identify novel therapeutic targets, and improve prognostic accuracy. ML and AI further facilitate integration of multidimensional data and the development of personalized treatment algorithms [281]. Pharmacogenomics shows promise for optimizing therapy—particularly for beta-blockers, ACEis, and SGLT2is—although clinical implementation remains limited by the need for robust validation and standardized methodologies [281]. The gut microbiome is increasingly recognized as a modifiable contributor to HF pathophysiology and a potential therapeutic target. Despite substantial progress, broad adoption of precision medicine faces challenges related to data standardization, cost-effectiveness, and clinical integration, underscoring the need for continued validation through prospective trials [281,282].

## 30. Gene and Cell-Based Therapies in Heart Failure

Gene and cell-based therapies remain investigational approaches for patients with advanced or refractory HF in whom conventional treatments are insufficient. Gene therapy has focused on correcting molecular abnormalities in cardiomyocytes, including enhancing calcium handling (e.g., SERCA2a, adenylyl cyclase 6), promoting angiogenesis, and modulating apoptosis and oxidative stress. Although early-phase trials demonstrated safety and preliminary efficacy, larger randomized studies such as CUPID-2 failed to show significant clinical benefit, underscoring ongoing challenges in vector delivery, transduction efficiency, and patient selection [283,284,285]. Cell-based therapies, particularly mesenchymal stromal cells derived from bone marrow, umbilical cord, or adipose tissue, have shown encouraging signals in phase I–II trials in both ischemic and nonischemic HF, with improvements in cardiac function and symptoms, likely mediated through paracrine mechanisms rather than direct cardiomyocyte replacement. Allogeneic cell products and repeat or intravenous dosing strategies are emerging as promising approaches. While major safety concerns have not been identified, definitive efficacy awaits confirmation in large phase III trials [286,287,288]. Accordingly, both gene and cell-based therapies are not recommended for routine HF management at present but remain important areas of ongoing investigation [287].

## 31. Challenges in Heart Failure Management

Despite significant advancements, several challenges persist:

1. HFpEF Therapies: HFpEF is particularly challenging due to its multifactorial etiology and multiple comorbidities, making diagnosis and management complex [10,289]. Due to the limited pharmacologic options currently available, novel therapeutic strategies tailored to specific HFpEF sub-phenotypes are needed [289].
2. Comorbidities: Effective and safe treatment options for HF patients with comorbid conditions such as CKD, Diabetes Mellitus, chronic lung disease, depression, cognitive disorders, and iron deficiency remain a significant challenge [1].
3. Advanced Disease Management: Interventional and device-based therapies, such as MCS devices, percutaneous mitral valve repair, and atrial fibrillation ablation, are being studied for their efficacy in advanced HF management. However, these therapies’ high complication rates and costs remain significant barriers to widespread clinical use [290,291].
4. Clinical Trial Representation: Special populations, including older adults, women, and minorities, remain underrepresented in clinical trials, requiring attempts to increase the diversity in study cohorts [1].
5. Disparities in Access: Addressing the social determinants of health and healthcare disparities is crucial. Strategies to eliminate disparities in healthcare access and socioeconomic factors are needed [291].
6. Integration of Technology: Genetic evaluation, biomarker-guided management, and remote monitoring technologies, such as wearable devices, telehealth, and AI integration, can provide personalized patient-centered care, potentially improving overall outcomes [291,292].
7. Long-Term Outcomes: While new therapies show promising short-term outcomes, data regarding their impact on long-term survival and quality of life warrant further investigation [1].

## 32. Conclusions

Significant advancements in HF diagnostics and therapeutics have emerged, with novel biomarkers, advanced imaging modalities, and pharmacological and device-based interventions reshaping HF management. However, challenges such as disparities in healthcare access and limited therapeutic options for HFpEF and advanced HF subtypes need to be addressed. A multidisciplinary, patient-centered approach is essential to improve patient outcomes and reduce the global burden of HF.

## Figures and Tables

**Table 1:** Summary of Emerging Biomarkers in Heart Failure and Their Clinical Utility.

Biomarker	Pathophysiological Role	Clinical Utility	Notes
sST2 (Soluble ST2)	Myocardial fibrosis, systemic inflammation	Risk stratification, prognostic assessment	Independent predictor of mortality; adds value beyond natriuretic peptides
Galectin-3 (Gal-3)	Myocardial fibrosis, inflammation	Risk stratification, prognostic assessment	Associated with adverse outcomes in both acute and chronic HF
GDF-15	Oxidative stress, myocardial inflammation	Mortality prediction, especially in acute HF	May exceed natriuretic peptides in prognostic accuracy
suPAR	Systemic inflammation	Prognostic marker, renal function assessment	Elevated levels associated with poor outcomes
MR-proADM	Neurohormonal activation	Prognostic marker	Reflects hemodynamic stress; complements natriuretic peptides
Cystatin C (CysC)	Renal dysfunction	Prognostic marker, renal function assessment	Useful in HF with comorbid kidney disease
High-sensitivity Troponins	Myocardial injury	Diagnosis and risk stratification	Remain standard; incremental value with other biomarkers
H-FABP	Myocardial injury	Early marker of myocardial stress	Rapidly released; complements troponins
VCAM-1	Endothelial dysfunction, inflammation	Emerging role in vascular remodeling and inflammation	Limited current clinical use
Copeptin	Neurohormonal activation (vasopressin pathway)	Prognostic marker	Reflects endogenous stress response
IL-6	Inflammation, adverse remodeling	Prognostic marker, reflects disease severity	Elevated in more symptomatic or severe HF
TNF-α	Inflammation, adverse cardiac remodeling	Prognostic marker, reflects disease progression	Associated with worse hemodynamics and poor outcomes

HF: Heart failure; sST2: Soluble suppression of tumorigenicity 2; Gal-3: Galectin-3; GDF-15: Growth differentiation factor-15; suPAR: Soluble urokinase-type plasminogen activator receptor; MR-proADM: Mid-regional pro-adrenomedullin; CysC: Cystatin C; H-FABP: Heart-type fatty acid-binding protein; VCAM-1: Vascular cell adhesion molecule 1; IL-6: Interleukin-6; TNF-α: Tumor necrosis factor-alpha.

**Table 5:** Heart Failure Management in Special Populations.

Population	Key Challenges	Guideline/Management Recommendations	Therapeutic Considerations and Evidence
Elderly (≥75–80 years)	- High prevalence of comorbidities (CKD, DM, cognitive impairment) - Frailty (up to 60%)- Polypharmacy - Altered PK/PD, organ function decline	- Initiate GDMT at lower doses with slow titration - Individualized care with close monitoring for adverse effects - Consider patient goals, functional and social status	- SGLT2i, ARNIs beneficial (improved LVEF, ↓NT-proBNP, ↓hospitalizations, ↓mortality) - Risks: hypotension, renal dysfunction, volume depletion, GU infections, fractures, ketoacidosis- Polypharmacy ↑ risks: renal dysfunction, electrolyte disorders, falls - Rehabilitation (multidomain, cardiac rehab) improves function, QoL, reduces rehospitalizations
Pregnancy and Peripartum Cardiomyopathy (PPCM)	- Symptom overlaps with pregnancy - Risk of severe HF and adverse maternal/fetal outcomes - Genetic predisposition (e.g., titin variants in ~20%)	- Early diagnosis with echo + natriuretic peptides - Multidisciplinary care (cardiology, MFM, genetics, neonatology) - Genetic counseling/testing recommended	- Avoid ACEi, ARBs, ARNIs, MRAs, SGLT2i (teratogenic) - Preferred: hydralazine + nitrates, metoprolol, cautious diuretics - Anticoagulation if LVEF < 30% until 6–8 wks postpartum - Bromocriptine (2.5 mg BID) in acute severe PPCM (“BOARD” regimen); requires prophylactic anticoagulation - MCS (ECMO, VAD) for refractory cases- Long-term: HF therapy, echo follow-up, contraceptive planning
HF with Diabetes Mellitus	- High cardiovascular risk - Overlap of DM and HF pathophysiology	- SGLT2i as first-line therapy for DM with HF or high HF risk - Use independent of baseline glycemic control	- SGLT2i (canagliflozin, dapagliflozin, empagliflozin, sotagliflozin) ↓MACE, HF hospitalizations, CV death - Greatest benefit in year 1 and high-risk patients - Monitor for GU infections, rare ketoacidosis
HF with Chronic Kidney Disease (CKD)	- HF and CKD are bidirectionally linked - Limited evidence in advanced CKD	- Use SGLT2i, ACEi/ARB, ARNI if eGFR ≥ 20 mL/min/1.73 m^2^ - Avoid premature discontinuation despite initial eGFR drop - Encourage combined cardio-nephrology care	- Proven ↓ HF hospitalization, ↓ CV death, ↓ CKD progression in mild–moderate CKD - Limited data in stage 4–5 CKD
HF with Autoimmune Disease (e.g., systemic sclerosis)	- Primary cardiac involvement common, poor prognosis - Limited trial evidence for HF therapies	- Multidisciplinary approach (cardiology + rheumatology)	- No disease-specific HF therapy - Observational data: immunosuppression may ↓ myocardial inflammation and improve biomarkers - Need larger prospective trials

ACC: American College of Cardiology; ACEi: angiotensin-converting enzyme inhibitor; ARB: angiotensin receptor blocker; ARNI: angiotensin receptor–neprilysin inhibitor; BNP: B-type natriuretic peptide; CKD: chronic kidney disease; CV: cardiovascular; DM: diabetes mellitus; ECMO: extracorporeal membrane oxygenation; eGFR: estimated glomerular filtration rate; GDMT: guideline-directed medical therapy; GU: genitourinary; HF: heart failure; HFpEF: heart failure with preserved ejection fraction; HFrEF: heart failure with reduced ejection fraction; LVEF: left ventricular ejection fraction: MAC: major adverse cardiovascular events; MCS: mechanical circulatory support; MRA: mineralocorticoid receptor antagonist; NT-proBNP: N-terminal pro-B-type natriuretic peptide; PPCM: peripartum cardiomyopathy; PK/PD: pharmacokinetics/pharmacodynamics; QoL: quality of life; SGLT2i: sodium-glucose cotransporter-2 inhibitor; VAD: ventricular assist device.

## Data Availability

All data used in this review article are from published content. Please see the reference lists.

## Abbreviations

The following abbreviations are used in this manuscript:

ACC	American College of Cardiology
ACEi	Angiotensin-Converting Enzyme Inhibitor
ACCP	American College of Chest Physicians
ACEP	American College of Emergency Physicians
AF	Atrial Fibrillation
AHA	American Heart Association
AL	Amyloid Light-chain
ARNI	Angiotensin Receptor–Neprilysin Inhibitor
ARB	Angiotensin Receptor Blocker
AS	Aortic Stenosis
ATTR-CM	Transthyretin Amyloid Cardiomyopathy
AUC	Area Under the Curve
BAT	Baroreflex Activation Therapy
BNP	B-type Natriuretic Peptide
BP	Blood Pressure
CAD	Coronary Artery Disease
CCM	Cardiac Contractility Modulation
CKD	Chronic Kidney Disease
CIED	Cardiac Implantable Electronic Device
CMR	Cardiac Magnetic Resonance Imaging
CRT	Cardiac Resynchronization Therapy
CCTA	Coronary Computed Tomography Angiography
CysC	Cystatin C
CV	Cardiovascular
cGMP	Cyclic Guanosine Monophosphate
DECT	Dual-Energy Computed Tomography
ECG	Electrocardiogram
ECV	Extracellular Volume
EF	Ejection Fraction
ESC	European Society of Cardiology
FoCUS	Focused Cardiac Ultrasound
GDF-15	Growth Differentiation Factor-15
GDMT	Guideline-Directed Medical Therapy
GLP-1RA	Glucagon-Like Peptide-1 Receptor Agonist
GLS	Global Longitudinal Strain
HCM	Hypertrophic Cardiomyopathy
HF	Heart Failure
HFpEF	Heart Failure with Preserved Ejection Fraction
HFrEF	Heart Failure with Reduced Ejection Fraction
HFmrEF	Heart Failure with Mildly Reduced Ejection Fraction
HFimpEF	Heart Failure with Improved Ejection Fraction
HFA	Heart Failure Association
hs-cTn	High-Sensitivity Cardiac Troponin
HR	Heart Rate
ICD	Implantable Cardioverter-Defibrillator
IL-6	Interleukin-6
IVC	Inferior Vena Cava
IVUS	Intravascular Ultrasound
KCCQ	Kansas City Cardiomyopathy Questionnaire
KEF	Kinetic Energy Fluctuation
LGE	Late Gadolinium Enhancement
LV	Left Ventricle
LVAD	Left Ventricular Assist Device
LUS	Lung Ultrasound
LVEF	Left Ventricular Ejection Fraction
MI	Myocardial Infarction
MIBG	Metaiodobenzylguanidine
MR-proADM	Mid-Regional Pro-Adrenomedullin
MRI	Magnetic Resonance Imaging
MRA	Mineralocorticoid Receptor Antagonist
NO	Nitric Oxide
NP	Natriuretic Peptide
NT-proBNP	N-terminal pro-B-type Natriuretic Peptide
NYHA	New York Heart Association
O_2_	Oxygen
PA	Pulmonary Artery
PARADIGM-HF	Prospective Comparison of ARNI with ACEi to Determine Impact on Global Mortality and Morbidity in Heart Failure
PET	Positron Emission Tomography
POCUS	Point-of-Care Ultrasound
PPG	Photoplethysmography
PYP	Technetium-99m Pyrophosphate
QoL	Quality of Life
RAAS	Renin–Angiotensin–Aldosterone System
RV	Right Ventricle
sGC	Soluble Guanylate Cyclase
sST2	Soluble Suppression of Tumorigenicity 2
SGLT2i	Sodium–Glucose Co-Transporter-2 Inhibitor
SNS	Sympathetic Nervous System
SPECT	Single-Photon Emission Computed Tomography
STE	Speckle Tracking Echocardiography
SU	Soluble Urokinase-Type Plasminogen Activator Receptor (suPAR)
TAVR	Transcatheter Aortic Valve Replacement
TEER	Transcatheter Edge-to-Edge Repair
TNF-α	Tumor Necrosis Factor-Alpha
VO_2_	Oxygen Consumption
VCAM-1	Vascular Cell Adhesion Molecule-1
